# A cross-sectional study from NHANES found a positive association between obesity with bone mineral density among postmenopausal women

**DOI:** 10.1186/s12902-023-01444-w

**Published:** 2023-09-13

**Authors:** Yu Yuan, Jiaxin Liao, Zhiyuan Luo, Dingshuang Li, Lei Hou

**Affiliations:** 1 The Eighth Clinical Medical College of Guangzhou University of Chinese Medicine, Foshan, Guangdong China; 2 Foshan Hospital of Traditional Chinese Medicine, Foshan, Guangdong China

**Keywords:** Postmenopausal women, Obesity, Positive relationship, Saturation value

## Abstract

**Purpose:**

Obesity has been demonstrated to improve bone mineral density (BMD), according to previous research. Nevertheless, there is a dearth of clarity regarding the optimal body mass index (BMI) and waist circumference (WC) for achieving the highest beneficial BMD in postmenopausal women. The objective of this study was to establish the correlation between obesity and BMD.

**Methods:**

The relationship between BMI, WC, and BMD was examined by using multivariate logistic regression models, fitting smoothing curves and utilizing the latest data from the National Health and Nutrition Examination Survey (NHANES) survey conducted between 2007 and 2018. Furthermore, the analysis of saturation effects was employed to examine the association of nonlinear connections among BMI, WC, and BMD.

**Results:**

The research examined information from a combination of 564 participants. A significant correlation between BMD and BMI as well as WC was observed in our findings. The enduring correlation between BMI and WC with BMD was demonstrated across subgroup analyses categorized by age and race, except among other Hispanic and other race. Furthermore, the smoothing curve fitting indicated that there existed not just a linear correlation among BMI, WC, and BMD, but also a saturation threshold in the association of these three factors.

**Conclusions:**

Based on our study, we have found a strong and positive relationship between obesity and BMD. According to the results of this research, maintaining obesity at a moderate level in postmenopausal women would result in achieving an optimal equilibrium between obesity and BMD.

**Supplementary Information:**

The online version contains supplementary material available at 10.1186/s12902-023-01444-w.

## Introduction

Osteoporosis, as a result of reduction of bone mineral density (BMD) and increase of bone fragility, causes the occurrence of fractures throughout the body [1]. It has become an increasingly significant global concern in terms of public health [1, 2]. According to previous research, around 20% of males and 33% of females who are 50 years old or older are susceptible to osteoporosis on a global scale [3]. Annually, over 10 million individuals in the United States experience osteoporosis, with the majority of cases affecting women after menopause [4, 5]. Hence, a large portion of individuals afflicted with osteoporosis are poised to achieve unprecedented accomplishments in the coming years.

Obesity, excessive fat accumulation status that may damage health, progressively evolved into a critical risk to community health [6]. Body mass index (BMI) [7] and waist circumference (WC) [8] are the most frequently accustomed to define obesity. Studies have demonstrated obesity is strongly and closely related to BMD. The pairing of BMI and WC could potentially provide new opportunities for preventing negative outcomes associated with obesity [9]. According to a study on Mendelian randomization involving 336,107 participants, Song [10] found that a higher BMI has an effect on increasing BMD, with consistent impacts observed throughout the skeleton. After adjusting all relevant variables, there were particular to age, gender and BMI correlations between WC and BMD [9].

The impact of obesity on BMD and the presence of saturation effect have been studied in older people [11] and adolescents [12]. However, BMD is strongly associated with postmenopausal women [13, 14]. There has been limited research on the relationship between obesity and BMD, as well as the presence of saturation effects, particularly in postmenopausal [15]. Given this context, our objective was to investigate potential connections between obesity and BMD, including the impact of saturation, among postmenopausal women. According to our research, maintaining the highest possible BMI and WC values would achieve the optimal equilibrium between obesity and BMD.

## Material and methods

### Study materials

All available information in the present study were acquired from The National Health and Nutrition Examination Survey (NHANES). During the years 2007 to 2018, data extracted from the NHANES was utilized for this inquiry. The conditions for inclusion were as follows: (a) individuals who have reached menopause and are at least 50 years of age, and (b) individuals with complete data, including BMD, BMI and WC. The following were the conditions for exclusion: (a) individuals with insufficient information on variables; (b) individuals with the history of female hormone or prednisone or cortisone use; (c) individuals who had the history of hysterectomy and then (d) individuals who had the history of hip or spine fracture. Finally, prior to being questioned and examined, all individuals participating in the survey provided their informed consent, which was approved by the Ethics Review Board of the National Center for Health Statistics.

### BMD testing and assessment

All subjects underwent whole-body scans using dual-energy X-ray absorptiometry (DXA). To conduct this study, BMD measurements were taken using Hologic QDR-4500A fan-beam densitometers (Hologic, Inc., Bedford, Massachusetts) during the period of 2017 to 2018. During the period of 2007 to 2016, the femur and spine were scanned using the Hologic Horizon Model A densimeter (manufactured by Hologic, Inc., located in Bedford, Massachusetts). In conclusion, the assessment encompassed the evaluation and inclusion of total spine BMD (LS-BMD), total femur BMD (TF-BMD), and femoral neck BMD (NK-BMD). Additional information can be found on the NHANES website [16].

### Obesity evaluation and grouping

The BMI was calculated by dividing the weight (kg) by the square of the height (m). Three categories were established: normal (25 kg/m^2^), overweight (25 ~ 30 kg/m^2^), and obese (> 30 kg/m^2^). To measure the WC, the right midaxillary line was obtained by drawing a horizontal line above the outermost edge of the right ilium and placing a tape measure at the point where the two lines intersected. At the junction of the two lines, a tape measure needs to be positioned. Finally, at the end of the individual’s normal expiration, his or her WC was measured. WC of 88 cm or more for women were termed abdominal obesity.

### Covariates

Taking into account the impact of additional factors on obesity and BMD, the chosen variables extracted from NHANES data include age, race, education level, alanine transaminase (ALT) and aspartate transaminase (AST), serum creatinine (SCr), 25OHD_2_ + 25OHD_3,_ total calcium and phosphorus, total cholesterol and triglyceride, history of smoking at least 100 cigarettes, diabetes and hypertension status, as well as duration of minutes sedentary activity.

## Statistical analysis

All analyses were conducted using the EmpowerStats (2.0) and R software (4.0.3). A p-valueis lower than 0.05 as the cutoff point for statistical significance. To showcase the fundamental characteristics, the baseline features are illustrated through the weighted mean ± standard error (SE) for continuous variables and the weighted proportion for categorical variables. To examine the linear correlation between obesity and BMD, a multivariate linear regression model with weights was employed. Subgroup analysis was conducted using multivariate linear regression models to determine the linear relationship between obesity and BMD among different age and race groups after stratifying the populations. Furthermore, the association between obesity and BMD was examined using generalized additive models and approximated smooth curves. By using a combination of two-piecewise linear regression models, the tipping point was determined.

## Results

### Baseline characteristics

**Fig. 1:**
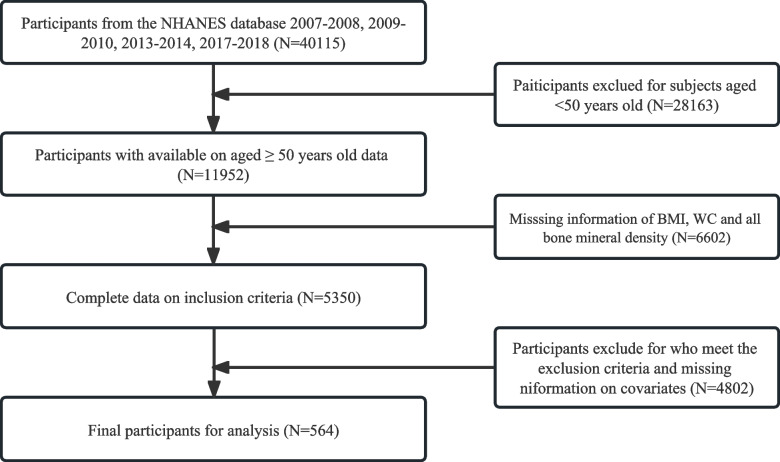
Flow diagram of the inclusion criteria and exclusion criteria

Figure 1 displays the information gathered on a total of 40,115 people from the NHANES dataset.

First of all, we excluded individuals aged less than 50 years (*N* = 28,163) and missing information of inclusion criteria (*N* = 6602). Moreover, participants who meet the exclusion criteria and missing information on variables were excluded (*N* = 6038). In the study, a grand total of 564 women aged 50 and over, who were postmenopausal, were included (Table 1).

**Table 1:** Weighted characteristics of the study population

	**Characteristics**	**Mean or proportion**
Age (year)		60.22 ± 8.59
Race, %	Mexican American	7.17
	Other Hispanic	6.13
	Non-Hispanic White	69.31
	Non-Hispanic Black	10.32
	Other Race	7.08
Education level, %	Less than high school	14.25
	High school	25.79
	More than high school	59.95
BMI (kg/m2)		28.08 ± 6.18
WC (cm)		95.83 ± 14.67
Smoked at least 100 cigarettes in life, %		
	Yes	34.86
	No	65.14
Diabetes, %		
	Yes	9.76
	No	87.48
	Borderline	2.76
Hypertension, %		
	Yes	38.53
	No	61.47
ALT (mmol/l)		21.22 ± 13.27
AST (mmol/l)		22.91 ± 8.61
SCr (mmol/l)		70.14 ± 28.19
Calcium (mmol/l)		2.34 ± 0.09
Phosphorus (mmol/l)		1.24 ± 0.16
Cholesterol (mmol/l)		5.41 ± 1.07
Triglyceride (mmol/l)		1.26 ± 0.75
25OHD2 + 25OHD3 (mmol/l)		74.23 ± 27.78
Minutes sedentary activity (min)		404.22 ± 632.54
TF-BMD (g/cm^2^)		0.86 ± 0.14
NK-BMD (g/cm^2^)		0.72 ± 0.13
LS-BMD (g/cm^2^)		0.95 ± 0.15

The weighted mean ± standard error (SE) (for continuous variables) and the weighted proportion (for categorical variables) serve to demonstrate the baseline features

*BMI* body mass index, *WC* waist circumfer-ence, *ALT* alanine transaminase, *AST* aspartate transaminase, *SCr* serum creatinine, *BMD* bonemineral density, *TF-BMD* total femur BMD, *NK-BMD* femoral neck BMD, *LS-BMD* total spine BMD

Overall, the average age of the participants was 60.22 ± 8.59 years, and the majority were non-Hispanic whites people (69.31%), followed by 10.32% non-Hispanic black people, 7.17% Mexican–American people, 6.13% other Hispanic people, and 7.08% individuals from diverse racial backgrounds. Furthermore, the average LS-BMD and TF-BMD, along with NK-BMD among all participants, were recorded as 0.95 ± 0.15 g/cm^2^, 0.86 ± 0.14 g/cm^2^, and 0.72 ± 0.13 g/cm^2^, respectively.

Supplementary Table 1 displays the clinical features of the subjects categorized by BMI as a column-stratified factor. There were no highly relevant differences in terms of age, education level, smoking statues, minutes sedentary activity, AST, SCr and total cholesterol. Obese individuals, in comparison to those who are normal weight or overweight, are more likely to have abdominal obesity, be non-Hispanic Caucasians, have diabetes and hypertension, and exhibit elevated levels of BMD, ALT, and total triglycerides. Additionally, they tend to have lower levels of 25OHD2 + 25OHD3, total calcium, and phosphorus.

Supplementary Table 2 displays the clinical traits of the subjects based on the WC. Participants who fell into the abdominal obesity tended to be non-Hispanic white, a BMI > 30 kg/m^2^, smokers, with no diabetes, hypertension, with higher BMD, ALT and total triglyceride and having poor levels of 25OHD2 + 25OHD3, total calcium and phosphorus.

### Associations between BMI and BMD

The correlation between BMI and BMD is outlined in Table 2. A favorable relationship between BMI and each BMD was discovered by using multivariate regression analysis. Furthermore, even after accounting for age and race in Model 2, the association between BMI and BMD remained positive. Meanwhile, When after adjusting for covariates in Model 3, the link remain stabled positively between exposed variables and outcomes. In addition, upon classifying BMI into different categories (Supplementary Table 1), the findings revealed a positive correlation between higher BMI and increased BMD among postmenopausal females. This association remained consistent across various BMI levels within the same group.

**Table 2:** Association between body mass index (kg/m^2^) and bone mineral density (g/cm^2^)

	**Model 1** **β (95% CI) ** ***P*** ** value**	**Model 2** **β (95% CI) ** ***P*** ** value**	**Model 3** **β (95% CI) ** ***P*** ** value**
**TF-BMD (g/cm**^**2**^**)**	0.01 (0.01, 0.01) < 0.0001	0.01 (0.01, 0.01) < 0.0001	0.01 (0.01, 0.01) < 0.0001
**NK-BMD (g/cm**^**2**^**)**	0.01 (0.01, 0.01) < 0.0001	0.01 (0.01, 0.01) < 0.0001	0.01 (0.01, 0.01) < 0.0001
**LS-BMD (g/cm**^**2**^**)**	0.01 (0.01, 0.01) < 0.0001	0.01 (0.01, 0.01) < 0.0001	0.01 (0.00, 0.01) < 0.0001

Model 1: non-adjusted model adjust none

Model 2: adjusted model adjust for age, race

Model 3: adjusted model adjust for age, race, education level, alanine transaminase (ALT) and aspartate transaminase (AST), serum creatinine (SCr), 25OHD2 + 25OHD3, total calcium and phosphorus, total cholestero-l and triglyceride, smoked at least 100 cigarettes in life, diabetes status, hypertension status andminutes sedentary activity

*BMI* body mass index, *BMD* bone mineral density, *TF-BMD* total femur BMD, *NK-BMD* femoral neck BMD, *LS-BMD* total spine BMD

On a subgroup analysis stratified by age (Table 3), the correlation between BMI and BMD was found in adults aged 50–59, 60–69, and over 70 years, which show a statistically positive correlation. In the subgroup analysis, when stratified by race (Table 3), the correlation between BMI and BMD remained significant in non-Hispanic black, Mexican–American, and other Hispanic individuals. Nevertheless, this correlation lost its significance in LS-BMD (*p* = 0.2608) when accounting for all other variables in non-Hispanic whites [0.00 (-0.00, 0.01)], the same as other race (*p* < 0.05).

**Table 3:** Association between body mass index (kg/m^2^) and bone mineral density (g/cm^2^) stratified age and race

Exposure	TF-BMD β (95% CI) *P* value	NK-BMD β (95% CI) *P* value	LS-BMD β (95% CI) *P* values
**Stratified by age**			
< 60	0.01 (0.01, 0.01) < 0.0001	0.01 (0.01, 0.01) < 0.0001	0.01 (0.00, 0.01) < 0.0001
≥ 60, < 70	0.01 (0.01, 0.01) < 0.0001	0.01 (0.00, 0.01) < 0.0001	0.01 (0.00, 0.01) < 0.0001
≥ 70	0.02 (0.01, 0.02) < 0.0001	0.01 (0.01, 0.02) < 0.0001	0.02 (0.01, 0.02) < 0.0001
**Stratified by race**			
Mexican American	0.01 (0.00, 0.01) 0.0001	0.01 (0.00, 0.01) 0.0003	0.01 (0.00, 0.01) 0.0004
Other Hispanic	0.01 (0.01, 0.02) < 0.0001	0.01 (0.01, 0.02) < 0.0001	0.00 (-0.00, 0.01) 0.2608
Non-Hispanic White	0.01 (0.01, 0.01) < 0.0001	0.01 (0.01, 0.01) < 0.0001	0.01 (0.00, 0.01) < 0.0001
Non-Hispanic Black	0.01 (0.00, 0.01) 0.0002	0.01 (0.00, 0.01) 0.0002	0.01 (0.00, 0.01) 0.0022
Other race	0.01 (0.00, 0.02) 0.0388	0.01 (-0.00, 0.02) 0.0597	0.00 (-0.01, 0.01) 0.4817

Adjusted for age, race, education level, alanine transaminase (ALT) and aspartate transaminase (AST), serum creatinine (SCr), 25OHD2 + 25OHD3, total calcium and phosphorus, total cholesterol and triglyceride,smoked at least 100 cigarettes in life, diabetes status, hypertension status and minutes sedentary activity

*BMD* bone mineral density, *TF-BMD* total femur BMD, *NK-BMD* femoral neck BMD, *LS-BMD* total spine BMD

**Fig. 2:**
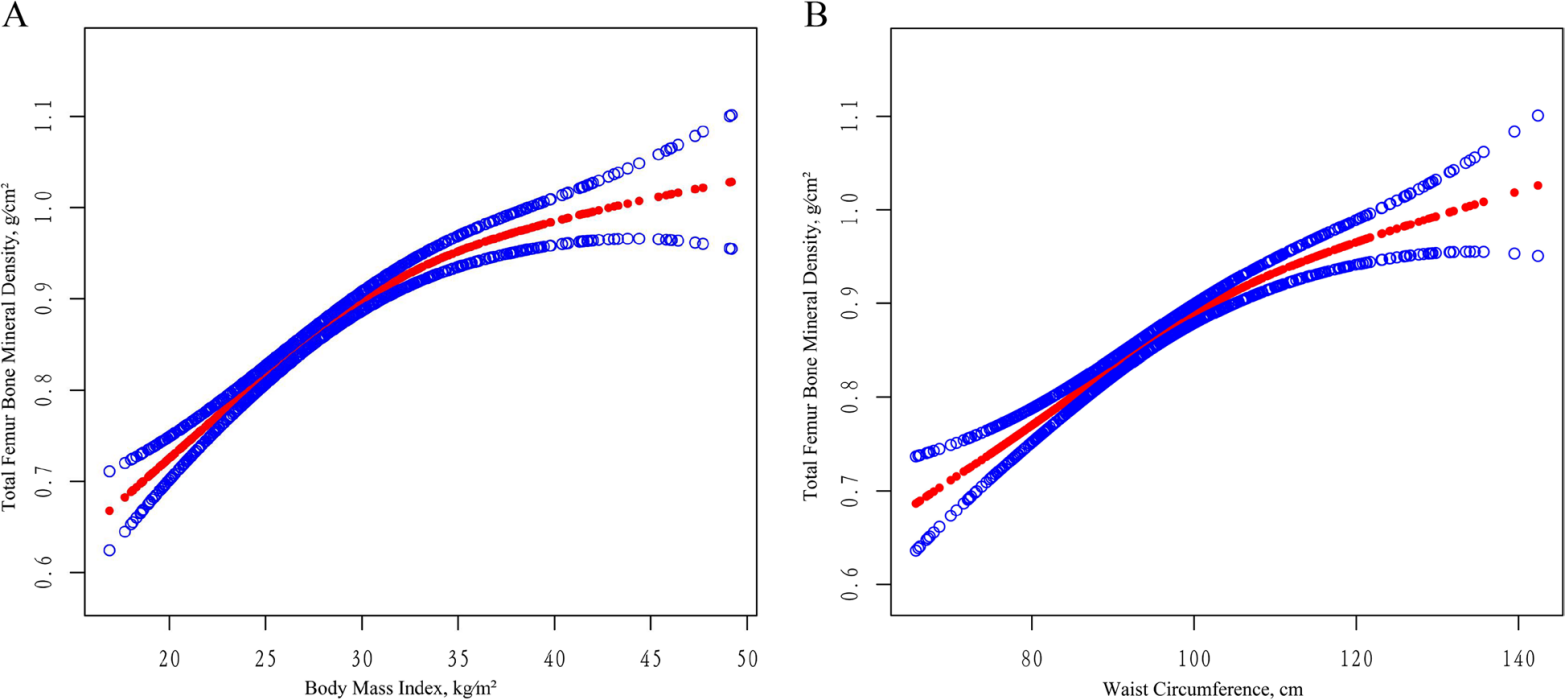
The association between obesity and total femural bone mineral density (g/cm^2^). **A** The association between body mass index and total femural bone mineral density. **B** The association between waist circumference and total femural bone mineral density. Adjusted for age, race, education level, alanine transaminase (ALT) and aspartate transaminase (AST), serum creatinine (SCr), 25OHD2 + 25OHD3, total calcium and phosphorus, total cholesterol and triglyceride,smoked at least 100 cigarettes in life, diabetes status, hypertension status and minutes sedentary activity

In the meantime, we employed the technique of curve smoothing to examine the association between BMI and BMD, which revealed a positive correlation (Fig. 2; Supplementary Fig. 1; Supplementary Fig. 2). In addition, with the help of the saturation effect analysis model, we discovered that the BMI threshold and saturation effect measure in TF-BMD, NK-BMD, and LS-BMD were 31.5 kg/m^2^, 31.5 kg/m^2^, and 30.1 kg/m^2^ correspondingly (Supplementary Table 5). The BMD increased in synchronize with each unit increase in BMI prior to the turning point. However, when the BMI surpassed the critical point, the increase in BMI per unit rise was negligible.

### Associations between WC with BMD

Table 4 exhibits the three distinct models of weighted multiple linear regression models. After controlling for confounding variables, a statistically positive connection between WC with BMD was revealed in three models. The correlation remained statistically significant even after stratifying WC (Supplementary Table 4). An increase of one unit in WC resulted in a significantly larger increase BMD compared to individuals with a WC less than 88 cm.

**Table 4:** Association between waist circumference (cm) and bone mineral density (g/cm^2^)

	**Model 1** **β (95% CI) ** ***P*** ** value**	**Model 2** **β (95% CI) ** ***P*** ** value**	**Model 3** **β (95% CI) ** ***P*** ** value**
**TF-BMD (g/cm**^**2**^**)**	0.00 (0.00, 0.00) < 0.0001	0.00 (0.00, 0.01) < 0.0001	0.00 (0.00, 0.01) < 0.0001
**NK-BMD (g/cm**^**2**^**)**	0.00 (0.00, 0.00) < 0.0001	0.00 (0.00, 0.00) < 0.0001	0.00 (0.00, 0.00) < 0.0001
**LS-BMD (g/cm**^**2**^**)**	0.00 (0.00, 0.00) < 0.0001	0.00 (0.00, 0.00) < 0.0001	0.00 (0.00, 0.00) < 0.0001

Model 1: non-adjusted model adjust none

Model 2: adjusted for age, race

Model 3: adjusted model adjust for age, race, education level, alanine transaminase (ALT) and aspartate transaminase (AST), serum creatinine (SCr), 25OHD2 + 25OHD3, total calcium and phosphorus, total cholesterol and triglyceride, smoked at least 100 cigarettes in life, diabetes status, hypertension status andminutes sedentary activity

*WC* waist circumfer-ence, *BMD* bone mineral density, *TF-BMD* total femur BMD, *NK-BMD* femoral neck BMD, *LS-BMD* total spine BMD

In the meantime, making use of generalized additive models (Supplementary Table 4) and smoothing curve fitting (Fig. 2; Supplementary Fig. 1; Supplementary Fig. 2), we identified a positive connection in the nonlinear relationship between WC and BMD. By employing the analysis model for saturation effect, we determined that the saturation effect values for TF-BMD, NK-BMD, and LS-BMD were 101.9 cm, 101.4 cm, and 103.2 cm, respectively (Supplementary Table 5). Before the tipping point, the BMD increased with each unit increase in WC, dramatically, while WC exceeded the tipping point, the rose only little per unit rise in BMD.

In the fully adjusted model, a positive association between BMD and WC was observed in the subgroup analysis based on age (*P* < 0.05) (Table 5). In the analysis of subgroups based on race, the findings were consistent with those of BMI and BMD, and the favorable correlation persisted among individuals of non-Hispanic black, Mexican–American, and other Hispanic backgrounds.Nevertheless, this correlation lost its significance in LS-BMD (*p* = 0.0745) when accounting for all other variables in non-Hispanic Caucasians [0.00 (-0.00, 0.01)].

**Table 5:** Association between waist circumference (cm) and bone mineral density (g/cm^2^) stratified age and race

Exposure	TF-BMD β (95% CI) *P* value	NK-BMD β (95% CI) *P* value	LS-BMD β (95% CI) *P* values
**Stratified by age**			
< 60	0.00 (0.00, 0.01) < 0.0001	0.00 (0.00, 0.00) < 0.0001	0.00 (0.00, 0.00) 0.0057
≥ 60, < 70	0.00 (0.00, 0.01) < 0.0001	0.00 (0.00, 0.00) < 0.0001	0.00 (0.00, 0.01) < 0.0001
≥ 70	0.01 (0.01, 0.01) < 0.0001	0.01 (0.00, 0.01) < 0.0001	0.01 (0.01, 0.01) < 0.0001
**Stratified by race**			
Mexican American	0.00 (0.00, 0.00) 0.0082	0.00 (0.00, 0.00) 0.0348	0.00 (0.00, 0.01) 0.0112
Other Hispanic	0.01 (0.00, 0.01) < 0.0001	0.01 (0.00, 0.01) < 0.0001	0.00 (-0.00, 0.01) 0.0745
Non-Hispanic White	0.00 (0.00, 0.01) < 0.0001	0.00 (0.00, 0.00) < 0.0001	0.00 (0.00, 0.00) < 0.0001
Non-Hispanic Black	0.00 (0.00, 0.01) 0.0003	0.00 (0.00, 0.01) 0.0011	0.00 (0.00, 0.01) 0.0033
Other race	0.00 (-0.00, 0.01) 0.0624	0.00 (-0.00, 0.01) 0.0991	0.00 (-0.00, 0.00) 0.5163

Adjusted for age, race, education level, alanine transaminase (ALT) and aspartate transaminase (AST), serum creatinine (SCr), 25OHD2 + 25OHD3, total calcium and phosphorus, total cholesterol and triglyceride,smoked at least 100 cigarettes in life, diabetes status, hypertension status and minutes sedentary activity

*BMD* bone mineral density, *TF-BMD* total femur BMD, *NK-BMD* femoral neck BMD, *LS-BMD* total spine BMD

## Discussion

The primary objective of this study was to investigate the association between obesity and BMD in postmenopausal women. We established a significant correlation between BMI, WC, and BMD in this research involving individuals above the age of 50. It is important to mention that there exists a saturation point connecting obesity and BMD, aligning with our prior hypothesis. BMD rose gradually as BMI or WC increased (at BMI or WC levels < turning point), while BMD roes gradually as they increase insignificantly (at BMI or WC levels > turning point).

Numerous studies have indicated a direct association BMI, WC, and BMD [17]. To date, the evidence regarding the connection has remained contentious. In middle-aged men with a BMI of at least 30 kg/m^2^ and women with a BMI below 25 kg/m^2^, a cross-sectional study discovered a negative correlation between WC and lumbar BMD [18]. This conclusion align with other studies [19–21]. Furthermore, several studies have indicated that WC was found to have a substantial positive relationship with total hip BMD, but a negative relationship with non-weight-bearing locationsn [22]. Possible causes for these variations may include demographic prejudice, the methodology employed for subgroup analysis, modifications in the criteria for inclusion or exclusion, discrepancies in skeletal characteristics across different locations, and the management of factors that may introduce bias. During the course of research, certain academics have come across an obesity paradox while utilizing BMI and WC as indicators of obesity [23, 24]. The presence of the obesity paradox prompts inquiries regarding the accuracy of BMI and WC as indicators of obesity, given their limitations in distinguishing between muscle mass and fat mass [25].

Although it is well proven that higher BMI or WC leads to a higher BMD, this does not mean that the risk of fracture is reduced. According to literature data, it has been suggested that obesity in postmenopausal females is linked to a higher likelihood of experiencing fractures in the ankle, other lower limbs, and humerus, while the risk of fractures in the hip and pelvis is reduced [26]. Regarding spinal fractures, the findings are considerably more conflicting [27]. Obese individuals may be shielded from pelvis and hip fractures by the presence of cushioning soft tissues. Individuals have a tendency to topple in a backward or sideways direction instead of forward, and combined with diminished defensive responses to falling, it could potentially safeguard the pelvis and hip from the force of the impact. However, the excess weight associated with obesity can potentially amplify the impact of a fall, surpassing the spine's capacity to handle it [26]. The causes of variations in fracture risk between obese and non-obese individuals at specific sites have not been determined yet. Maintaining a reasonable range of BMI and WC is important due to the reduced bone mass and heightened vulnerability to fractures linked with obesity.

Currently, the mechanism behind the link between obesity with BMD is complex and there were multiple mechanisms may exist. When in the event of excessive body fat accumulation and elevated obesity levels, it leads to an increase in static mechanical pressures on the skeleton, which in turn causes changes in bone structure [28]. In addition, mesenchymal stem cells (BMSCs)in the bone marrow give rise to osteoblasts and adipocytes [29]. Obesity augments the population of adipocytes within the bone marrow, lipogenic differentiation enhancing while osteogenic differentiation reducing [29]. In addition, obesity-induced hypermetabolism stimulates osteoblasts directly by increasing insulin signaling [30]. Furthermore, adipocytes can also release other hormones including adiponectin [31], leptin [32] and insulin [33], as well as adipocytic estrogens, all of which are beneficial to BMD by promoting bone remodelling and occupying bone resorption.

During this examination, we found that this correlation was altered based on ethnicity. Such individuals, who are other Hispanic and other races, should be alarmed. Sedentary activities are risk factors for obesity, however, it is consist with conclusions inversely [34]. Paradoxical outcomes could have been influenced by variations in study sample size, participant diversity, and the adjustment of covariation-adjusted heterogeneity. Additionally, obesity is associated with reduced levels of 25-hydroxyvitamin D. This can be explained by the storage of this vitamin in fatty tissue, although there is also proof suggesting that vitamin D hinders the formation of fat cells [33].

As individuals age, the arrangement of adipose tissue undergoes alterations. The rise in visceral fat, particularly, contributes to an elevated cardiovascular risk among elderly individuals and is additionally linked to a decline in BMD [35]. Furthermore, obesity is linked to a higher vulnerability to inflammation. This inflammatory condition also raises the chances of developing insulin resistance, type 2 diabetes, and arteriosclerosis, consequently elevating the likelihood of osteoporosis and fractures [23]. Smoking reveals a direct adverse effect on bone by activating nuclear factor kappa-B pathway, damaging collagen metabolism and impeding, bone angiogenesis [36]. Compared with individuals without diabetes, hypertension, and smoking, there is a strongly correlation between obesity and BMD.

We have discovered and verified the correlation between obesity and BMD saturation effect values. This phenomenon represents a unique bone–fat relationship that exists in vivo between adipose and bone tissue [37]. It is connected by a range of bioactive chemicals and plays a crucial role in maintaining bone homeostasis [37]. But further research is required to validate these findings, as a result, the reasons for the saturating effect of obesity on BMD remain to be not completely comprehended.

The results of our study have a high degree of generalizability due to the availability of data from a nationally representative sample in the NHANES survey. And it is important to recognize the constraints of our research as well. Firstly, due to the cross-sectional nature of this study, our findings only allow us to infer an association between obesity and BMD, rather than establishing a direct causal relationship. Secondly, we were unable to determine participants' menstrual age, menopausal phase, living habits, dietary patterns, bone metabolism indicators, and more, due to database constraints. Thirdly, the dearth of information about individuals' diseases information, it was impossible for us to identify those with a past medical history of thyroid disease, and metabolic disorders, leading to the inevitable problem of bone loss [1, 5]. Fourthly, there is a strong link between body composition with lean body mass, further when exploring the link between obesity and BMD, it is not possible to be overlooked. Lastly, based on our current understanding, the investigation was carried out on the US population, using a limited sample size, so the results should be approached with caution.

## Conclusion

Our research revealed a correlation and a point of saturation between obesity and BMD. This work suggested that the greatest advantage for women in terms of optimum BMD and reduction of other obesity-related disorders was achieved by maintaining a healthy obese value. Nevertheless, considering the inherent limitations imposed by the present investigation, further extensive research is necessary to validate these discoveries.

### Supplementary Information

**Additional file 1: Supplementary Table 1.** General characteristics of participants by body mass index (kg/m^2^).

**Additional file 2: Supplementary Table 2.** General characteristics of participants by waist circumference (cm).

**Additional file 3: Supplementary Table 3. **Association between body mass index (kg/m²) and bone mineral density (g/cm^2^).

**Additional file 4: Supplementary Table 4. **Association between waist circumference (cm) and bone mineral density (g/cm^2^).

**Additional file 5: Supplementary Table 5. **Saturation effect analysis of obesity on total BMD (g/cm^2^).

**Additional file 6: Supplementary Figure 1. **The association between obesity and femoral neck bone mineral density (g/cm^2^).

**Additional file 7: Supplementary Figure 2.** The association between obesity and total spine bone mineral density (g/cm^2^).

## Data Availability

The datasets supporting the conclusions of this article are available in the repository, (https://www.cdc.gov/nchs/nhanes/).
