# Supplementary material for: A cross-sectional study from NHANES found a positive association between obesity with bone mineral density among postmenopausal women
Source: BMC Endocr Disord. 2023 Sep 13;23:196. doi: 10.1186/s12902-023-01444-w (PMC10498604; doi:10.1186/s12902-023-01444-w)
Supplement: Supplementary file 1 — Additional file 1: Supplementary Table 1. General characteristics of participants by body mass index (kg/m2). [file 12902_2023_1444_MOESM1_ESM.docx]

SUPPLEMENTARY TABLE 1 | General characteristics of participants by body mass index (kg/m^2^).

|  | BMI (kg/m2) categorical | | | P-value |
| --- | --- | --- | --- | --- |
|  | BMI<25 | 25≤BMI<30 | BMI≥30 |  |
| Age (year) | 59.76 ± 8.56 | 61.23 ± 9.34 | 59.76 ± 7.75 | 0.1691 |
| Race, % |  |  |  | <0.0001 |
| Mexican American | 4.48 | 9.83 | 7.51 |  |
| Other Hispanic | 5.16 | 8.33 | 5.96 |  |
| Non-Hispanic White | 73.88 | 64.75 | 68.74 |  |
| Non-Hispanic Black | 4.09 | 9.61 | 17.77 |  |
| Other Race | 12.39 | 7.48 | 0.93 |  |
| Education level, % |  |  |  | 0.1390 |
| Less than high school | 10.62 | 18.97 | 13.66 |  |
| High school | 25.25 | 27.56 | 24.68 |  |
| More than high school | 64.13 | 53.47 | 61.66 |  |
| WC (cm) | 82.54 ± 7.09 | 93.97 ± 5.95 | 112.09 ± 10.39 | <0.0001 |
| Smoked at least 100 cigarettes in life, % |  |  |  | 0.0214 |
| Yes | 30.46 | 31.58 | 42.80 |  |
| No | 69.54 | 68.42 | 57.20 |  |
| Diabetes, % |  |  |  | <0.0001 |
| Yes | 3.65 | 11.61 | 14.60 |  |
| No | 95.65 | 86.77 | 79.30 |  |
| Borderline | 0.70 | 1.62 | 6.10 |  |
| Hypertension, % |  |  |  | <0.0001 |
| Yes | 24.52 | 39.17 | 53.15 |  |
| No | 75.48 | 60.83 | 46.85 |  |
| ALT (mmol/l) | 19.54 ± 7.85 | 20.99 ± 17.68 | 23.27 ± 12.73 | 0.0213 |
| AST (mmol/l) | 22.89 ± 6.92 | 22.55 ± 8.32 | 23.29 ± 10.34 | 0.7142 |
| SCr (mmol/l) | 71.21 ± 41.15 | 70.26 ± 18.47 | 68.87 ± 15.86 | 0.7176 |
| Calcium (mmol/l) | 2.34 ± 0.09 | 2.36 ± 0.10 | 2.33 ± 0.08 | <0.0001 |
| Phosphorus (mmol/l) | 1.30 ± 0.15 | 1.22 ± 0.14 | 1.18 ± 0.15 | <0.0001 |
| Cholesterol (mmol/l) | 5.34 ± 0.95 | 5.56 ± 1.19 | 5.34 ± 1.06 | 0.0802 |
| Triglyceride (mmol/l) | 1.02 ± 0.59 | 1.35 ± 0.69 | 1.42 ± 0.88 | <0.0001 |
| 25OHD2+25OHD3 (mmol/l) | 81.63 ± 27.63 | 72.30 ± 24.16 | 68.03 ± 29.32 | <0.0001 |
| Minutes sedentary activity (min) | 400.94 ± 204.65 | 403.75 ± 907.60 | 408.23 ± 617.59 | 0.9936 |
| TF-BMD (g/cm^2^) | 0.79 ± 0.11 | 0.86 ± 0.12 | 0.95 ± 0.13 | <0.0001 |
| NK-BMD (g/cm^2^) | 0.66 ± 0.11 | 0.72 ± 0.12 | 0.79 ± 0.12 | <0.0001 |
| LS-BMD (g/cm^2^) | 0.89 ± 0.13 | 0.95 ± 0.14 | 1.01 ± 0.15 | <0.0001 |

The weighted mean ± standard error (SE) (for continuous variables) and the weighted proportion (for

categorical variables) serve to demonstrate the baseline features. BMI, body mass index; WC, waist

circumference; ALT, alanine transaminase; AST, aspartate transaminas; SCr, serum creatinine; BMD, bone mineral density; TF-BMD, total femur BMD; NK-BMD, femoral neck BMD; LS-BMD, total spine BMD.
