# Supplementary material for: A cross-sectional study from NHANES found a positive association between obesity with bone mineral density among postmenopausal women
Source: BMC Endocr Disord. 2023 Sep 13;23:196. doi: 10.1186/s12902-023-01444-w (PMC10498604; doi:10.1186/s12902-023-01444-w)
Supplement: Supplementary file 2 — Additional file 2: Supplementary Table 2. General characteristics of participants by waist circumference (cm). [file 12902_2023_1444_MOESM2_ESM.docx]

SUPPLEMENTARY TABLE 2 | General characteristics of participants by waist circumference (cm).

|  | WC (cm) categorical | | P-value |
| --- | --- | --- | --- |
|  | <88 (cm) | >88 (cm) |  |
| Age (year) | 59.62 ± 8.73 | 60.51 ± 8.51 | 0.2537 |
| Race, % |  |  | <0.0001 |
| Mexican American | 4.17 | 8.57 |  |
| Other Hispanic | 6.14 | 6.12 |  |
| Non-Hispanic White | 72.31 | 67.89 |  |
| Non-Hispanic Black | 4.61 | 13.00 |  |
| Other Race | 12.76 | 4.41 |  |
| Education level, % |  |  | 0.0112 |
| Less than high school | 9.38 | 16.54 |  |
| High school | 22.15 | 27.50 |  |
| More than high school | 68.47 | 55.95 |  |
| BMI (kg/m2) | 22.41 ± 2.51 | 30.75 ± 5.56 | <0.0001 |
| Smoked at least 100 cigarettes in life, % |  |  | 0.0066 |
| Yes | 26.90 | 38.60 |  |
| No | 73.10 | 61.40 |  |
| Diabetes, % |  |  | 0.0006 |
| Yes | 4.17 | 12.38 |  |
| No | 95.27 | 83.83 |  |
| Borderline | 0.56 | 3.79 |  |
| Hypertension, % |  |  | 0.0005 |
| Yes | 0.0006 | 43.40 |  |
| No | 71.82 | 56.60 |  |
| ALT (mmol/l) | 18.84 ± 6.04 | 22.34 ± 15.42 | 0.0034 |
| AST (mmol/l) | 22.56 ± 5.75 | 23.08 ± 9.66 | 0.5064 |
| SCr (mmol/l) | 67.80 ± 12.97 | 71.24 ± 32.94 | 0.1772 |
| Calcium (mmol/l) | 2.35 ± 0.09 | 2.34 ± 0.09 | 0.3042 |
| Phosphorus (mmol/l) | 1.27 ± 0.15 | 1.22 ± 0.16 | 0.0012 |
| Cholesterol (mmol/l) | 5.46 ± 0.92 | 5.38 ± 1.14 | 0.4365 |
| Triglyceride (mmol/l) | 1.01 ± 0.51 | 1.38 ± 0.81 | <0.0001 |
| 25OHD2+25OHD3 (mmol/l) | 82.53 ± 26.76 | 70.33 ± 27.39 | <0.0001 |
| Minutes sedentary activity (min) | 398.36 ± 210.49 | 406.97 ± 753.16 | 0.8804 |
| TF-BMD (g/cm^2^) | 0.78 ± 0.12 | 0.90 ± 0.13 | <0.0001 |
| NK-BMD (g/cm^2^) | 0.66 ± 0.11 | 0.75 ± 0.13 | <0.0001 |
| LS-BMD (g/cm^2^) | 0.89 ± 0.13 | 0.98 ± 0.15 | <0.0001 |

The weighted mean ± standard error (SE) (for continuous variables) and the weighted proportion (for

categorical variables) serve to demonstrate the baseline features. WC, waist circumference; BMI, body

mass index; ALT, alanine transaminase; AST, aspartate transaminas; SCr, serum creatinine; BMD, bone

mineral density; TF-BMD, total femur BMD; NK-BMD, femoral neck BMD; LS-BMD, total spine BMD.
