# Supplementary material for: A cross-sectional study from NHANES found a positive association between obesity with bone mineral density among postmenopausal women
Source: BMC Endocr Disord. 2023 Sep 13;23:196. doi: 10.1186/s12902-023-01444-w (PMC10498604; doi:10.1186/s12902-023-01444-w)
Supplement: Supplementary file 3 — Additional file 3: Supplementary Table 3. Association between body mass index (kg/m²) and bone mineral density (g/cm2). [file 12902_2023_1444_MOESM3_ESM.docx]

SUPPLEMENTARY TABLE 3 | Association between body mass index (kg/m²) and bone mineral density (g/cm^2^).

|  | Model 1  β (95% CI) P value | Model 2  β (95% CI) P value | Model 3  β (95% CI) P value |
| --- | --- | --- | --- |
| TF-BMD (g/cm^2^) |  |  |  |
| <25 kg/m^2^ | Reference | Reference | Reference |
| ≥25 kg/m^2^, <30 kg/m^2^ | 0.07 (0.05, 0.10)  <0.0001 | 0.08 (0.06, 0.10)  <0.0001 | 0.08 (0.06, 0.11)  <0.0001 |
| ≥30 kg/m^2^ | 0.16 (0.14, 0.19)  <0.0001 | 0.16 (0.14, 0.18)  <0.0001 | 0.16 (0.14, 0.19)  <0.0001 |
| NK-BMD (g/cm^2^) |  |  |  |
| <25 kg/m^2^ | Reference | Reference | Reference |
| ≥25 kg/m^2^, <30 kg/m^2^ | 0.06 (0.04, 0.08)  <0.0001 | 0.07 (0.04, 0.09)  <0.0001 | 0.06 (0.04, 0.08)  <0.0001 |
| ≥30 kg/m^2^ | 0.13 (0.11, 0.15)  <0.0001 | 0.12 (0.10, 0.15)  <0.0001 | 0.12 (0.10, 0.15)  <0.0001 |
| LS-BMD (g/cm^2^) |  |  |  |
| <25 kg/m^2^ | Reference | Reference | Reference |
| ≥25 kg/m^2^, <30 kg/m^2^ | 0.06 (0.03, 0.09)  <0.0001 | 0.12 (0.10, 0.15)  <0.0001 | 0.06 (0.03, 0.09)  <0.0001 |
| ≥30 kg/m^2^ | 0.06 (0.03, 0.09)  <0.0001 | 0.12 (0.09, 0.15)  <0.0001 | 0.10 (0.07, 0.14)  <0.0001 |

Model 1: non-adjusted model adjust none.

Model 2: adjusted model adjust for age, race.

Model 3: adjusted model adjust for age, race, education level, alanine transaminase (ALT) and aspartate

transaminase (AST), serum creatinine (SCr), 25OHD2+25OHD3, total calcium and phosphorus, total

cholesterol and triglyceride, smoked at least 100 cigarettes in life, diabetes status, hypertension status andminutes sedentary activity. BMD, bone mineral density; TF-BMD, total femur BMD; NK-BMD, femoral

neck BMD; LS-BMD, total spine BMD.
