# Supplementary material for: A cross-sectional study from NHANES found a positive association between obesity with bone mineral density among postmenopausal women
Source: BMC Endocr Disord. 2023 Sep 13;23:196. doi: 10.1186/s12902-023-01444-w (PMC10498604; doi:10.1186/s12902-023-01444-w)
Supplement: Supplementary file 4 — Additional file 4: Supplementary Table 4. Association between waist circumference (cm) and bone mineral density (g/cm2). [file 12902_2023_1444_MOESM4_ESM.docx]

SUPPLEMENTARY TABLE 4 | Association between waist circumference (cm) and bone mineral density (g/cm^2^).

|  | Model 1  β (95% CI) P value | Model 2  β (95% CI) P value | Model 3  β (95% CI) P value |
| --- | --- | --- | --- |
| TF-BMD (g/cm^2^) |  |  |  |
| <88 cm | Reference | Reference | Reference |
| ≥88 cm | 0.12 (0.10, 0.14)  <0.0001 | 0.12 (0.10, 0.14)  <0.0001 | 0.12 (0.09, 0.14)  <0.0001 |
| NK-BMD (g/cm^2^) |  |  |  |
| <88 cm | Reference | Reference | Reference |
| ≥88 cm | 0.09 (0.07, 0.11)  <0.0001 | 0.09 (0.07, 0.11)  <0.0001 | 0.08 (0.06, 0.10)  <0.0001 |
| LS-BMD (g/cm^2^) |  |  |  |
| <88 cm | Reference | Reference | Reference |
| ≥88 cm | 0.09 (0.06, 0.11)  <0.0001 | 0.09 (0.06, 0.11)  <0.0001 | 0.08 (0.05, 0.10)  <0.0001 |

Model 1: non-adjusted model adjust none.

Model 2: adjusted model adjust for age, race.

Model 3: adjusted model adjust for age, race, education level, alanine transaminase (ALT) and aspartate

transaminase (AST), serum creatinine (SCr), 25OHD2+25OHD3, total calcium and phosphorus, total

cholesterol and triglyceride, smoked at least 100 cigarettes in life, diabetes status, hypertension status andminutes sedentary activity. BMD, bone mineral density; TF-BMD, total femur BMD; NK-BMD, femoral

neck BMD; LS-BMD, total spine BMD.
