# Supplementary material for: A cross-sectional study from NHANES found a positive association between obesity with bone mineral density among postmenopausal women
Source: BMC Endocr Disord. 2023 Sep 13;23:196. doi: 10.1186/s12902-023-01444-w (PMC10498604; doi:10.1186/s12902-023-01444-w)
Supplement: Supplementary file 5 — Additional file 5: Supplementary Table 5. Saturation effect analysis of obesity on total BMD (g/cm2). [file 12902_2023_1444_MOESM5_ESM.docx]

SUPPLEMENTARY TABLE 5 | Saturation effect analysis of obesity on total BMD (g/cm^2^).

|  | **TF-BMD (g/cm^2^)** | **NK-BMD (g/cm^2^)** | **LS-BMD (g/cm^2^)** |
| --- | --- | --- | --- |
| **BMI turning point, kg/m^2^** | 31.5 | 31.5 | 30.1 |
| < point | 0.02 (0.01, 0.02)  <0.0001 | 0.01 (0.01, 0.02)  <0.0001 | 0.01 (0.01, 0.02)  <0.0001 |
| > point | 0.00 (-0.00, 0.01)  0.1858 | 0.00 (-0.00, 0.01)  0.2799 | -0.00 (-0.00, 0.00)  0.8125 |
| P for log likelihood ratio test | <0.001 | <0.001 | <0.001 |
| **WC turning point, cm** | 101.9 | 101.4 | 103.2 |
| < point | 0.01 (0.01, 0.01)  <0.0001 | 0.00 (0.00, 0.01)  <0.0001 | 0.00 (0.00, 0.01)  <0.0001 |
| > point | 0.00 (0.00, 0.00)  0.0026 | 0.00 (-0.00, 0.00)  0.0652 | 0.00 (-0.00, 0.00)  0.6227 |
| P for log likelihood ratio test | <0.001 | <0.001 | 0.003 |

Adjusted for age, race, education level, alanine transaminase (ALT) and aspartate transaminase (AST),

serum creatinine (SCr), 25OHD2+25OHD3, total calcium and phosphorus, total cholesterol and triglyceride,smoked at least 100 cigarettes in life, diabetes status, hypertension status and minutes sedentary activity.

BMI, body mass index; WC, waist circumference; BMD, bone mineral density; TF-BMD, total femur

BMD; NK-BMD, femoral neck BMD; LS-BMD, total spine BMD.
