# Supplementary material for: A cross-sectional study from NHANES found a positive association between obesity with bone mineral density among postmenopausal women
Source: BMC Endocr Disord. 2023 Sep 13;23:196. doi: 10.1186/s12902-023-01444-w (PMC10498604; doi:10.1186/s12902-023-01444-w)
Supplement: Supplementary file 6 — Additional file 6: Supplementary Figure 1. The association between obesity and femoral neck bone mineral density (g/cm2). [file 12902_2023_1444_MOESM6_ESM.pdf]

SUPPLEMENTARY FIGURE 1 | The association between obesity and femoral neck bone mineral density (g/cm^2).

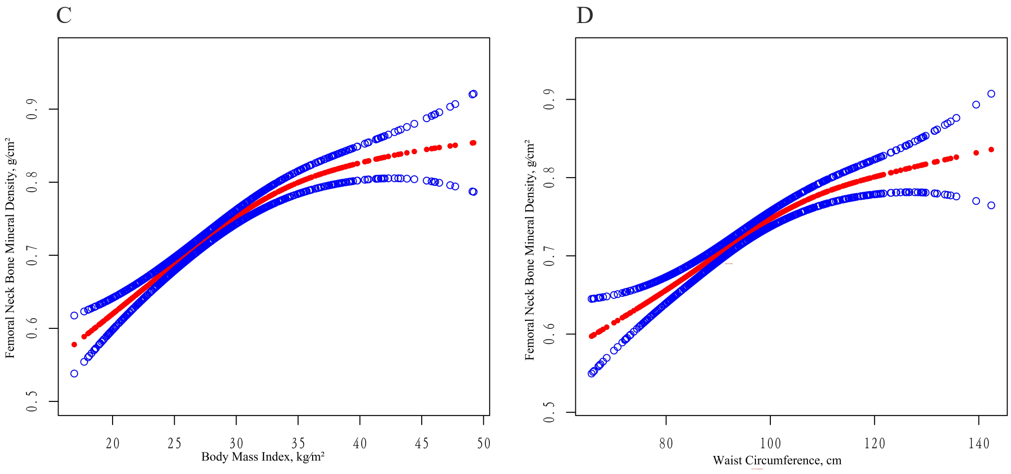

C: The association between body mass index and femoral neck bone mineral density.

D: The association between waist circumference and femoral neck bone mineral density.

Adjusted for age, race, education level, alanine transaminase (ALT) and aspartate transaminase (AST), serum creatinine (SCr), 25OHD2+25OHD3, total calcium and phosphorus, total cholesterol and triglyceride, smoked at least 100 cigarettes in life, diabetes status, hypertension status and minutes sedentary activity.
